# *Pythium huanghuaiense* sp. nov. isolated from soybean: morphology, molecular phylogeny and pathogenicity

**DOI:** 10.3897/BDJ.9.e65227

**Published:** 2021-04-22

**Authors:** Jia-Jia Chen, Hui Feng, Jian Yu, Wenwu Ye, Xiaobo Zheng

**Affiliations:** 1 College of Landscape Architecture, Jiangsu Vocational College of Agriculture and Forestry, Zhenjiang 212400, China College of Landscape Architecture, Jiangsu Vocational College of Agriculture and Forestry Zhenjiang 212400 China; 2 Department of Plant Pathology, Nanjing Agricultural University, Nanjing 210095, China Department of Plant Pathology, Nanjing Agricultural University Nanjing 210095 China

**Keywords:** Cox1, ITS, oomycete, *Pythium* clade F

## Abstract

**Background:**

Soybean (*Glycine max*) is a major source of edible oil and protein. A novel species of the genus *Pythium*, *Pythium huanghuaiense*, isolated from soybean seedlings in China, is described and illustrated on the basis of morphological characters and molecular evidence.

**New information:**

*Pythium huanghuaiense* sp. nov. is closely related to species of the genus *Pythium* in clade F, as evidenced by the presence of hyphal swellings and its relatively rapid morphological growth. However, it differs by having relatively small sporangia and plerotic or nearly plerotic and thin-walled oospores. A pathogenicity test confirmed the newly-identified species as a pathogen of soybean.

## Introduction

Species of the genus *Pythium* Pringsheim are diverse, occupying a variety of habitats (van der Plaäts-Niterink 1981). The genus was established by Pringsheim (1858), based on *Pythium monospermum* Pringsh. and is characterised by globose, oval, ellipsoidal, elongated, filamentous or toruloid sporangia and the development of zoospores in a vesicle formed at the tip of a discharge tube derived from a sporangium (van der Plaäts-Niterink 1981). There are more than 160 species of *Pythium* (Long et al. 2012, Long et al. 2014, Uzuhashi et al. 2015, Ueta and Tojo 2016, Chen et al. 2020), which includes many important plant pathogens that frequently cause seed, seedling and root rot in economically-important crops, such as soybean (*Glycine max*), wheat (*Triticum* spp.) and corn (*Zea mays*) (Wang et al. 2003, Wrather and Koenning 2006). Some *Pythium* spp. are important pathogens of animals, while others are beneficial as biological control agents that protect against pathogenic fungi (van der Plaäts-Niterink 1981, Ali-Shtayeh and Saleh 1999). To date, 74 species of *Pythium* have been reported in China (Ho 2013, Long et al. 2014, Chen et al. 2020).

Huang-Huai Valley is one of the main areas of soybean farming in China, covering an enormous area in Shandong, Anhui, Jiangsu and Henan Provinces between the Yellow River and the Haihe River. During the studies on the diversity of *Pythium* in the Huang-Huai Valley, a novel species of clade F was identified, based on morphological characters and molecular phylogenetic analyses of internal transcribed spacer (ITS) and cytochrome c oxidase subunit I (Cox1) sequence data. The novel species is described and illustrated in this work. Moreover, comparisons of the novel species with morphologically and phylogenetically related species are also provided.

## Materials and methods

### Isolates

During April and August 2016, 60 plants of soybean cultivar 'Hefeng 47' exhibiting seedling blight, damping off and root rot were collected from three fields in the Huang-Huai region of China. 'Hefeng 47' is commonly grown in the Huang-Huai Valley. The fields were located in Jining of Shandong Province, Suzhou of Anhui Province and Nanjing of Jiangsu Province, which are representative geographic locations in the Huang-Huai region. Soybean plants were sampled from fields at approximately 10 m intervals along a 150 m transect laid out in a “W” pattern.

Soybean plants were washed three times with sterile water and six sections of 0.5–1 cm length were cut from the roots of each plants using a sterile scalpel. One section was taken from the root tip, one from the interface between the hypocotyl and soil and the others at either the middle of the root or a symptomatic area along the length of the root. The sections were blotted dry and embedded in selective V8 juice agar (V8A) containing rifampicin (50 mg/l), ampicillin (50 mg/l) and pentachloronitrobenzene (50 mg/l) and incubated for 2–3 days in the dark at 25°C. When mycelial growth was observed, cultures were purified by transferring a small piece of medium with mycelium at the edge of a colony to fresh medium or by transferring a single hyphal tip on to water agar three times.

### Morphology and growth rate

The cultures studied were deposited in the Herbaria of the Institute of Microbiology, Beijing Forestry University (BJFC), Beijing, China; the College of Plant Protection, Nanjing Agricultural University (NJAU), Nanjing, China; and the College of Landscape Architecture, Jiangsu Vocational College of Agriculture and Forestry (JAFLA), Zhenjiang, China. Purified isolates were examined after incubation for 2–3 days at 25°C on V8A in the dark. Colony patterns of the representative isolate of the novel species were examined after incubation for 3 days at 25°C on corn meal agar (CMA), potato carrot agar (PCA) and V8A media (Miller 1955, van der Plaäts-Niterink 1981). Isolates were transferred to sterilised distilled water to induce sporulation. Fifty measurements were taken for each morphological feature, such as sporangia, oogonia and oospores. Cardinal temperatures were examined on PCA as described by van der Plaäts-Niterink (1981) and growth rates were measured after 24 h of incubation. Each isolate was incubated on PCA media at 5–40°C with intervals of 5°C. When no growth was observed, the intervals were reduced from 5°C to 2°C or 1°C and the culture was returned to room temperature to ensure that the strain could start growing again. The experiment was repeated twice using a single plate per repetition.

### Molecular phylogeny

**DNA extraction, amplification, sequencing and sequence alignment**

A cetyl trimethylammonium bromide (CTAB) rapid plant genome extraction kit (FH Plant DNA kit, Demeter Biotechnologies Co. Ltd, Beijing, China) was used to extract total genomic DNA from purified isolates and the polymerase chain reaction (PCR) was performed according to the manufacturer’s instructions (Cui et al. 2019). PCR amplification was carried out in 30-μl volumes consisting of 1 μl of DNA template, 1 μl of each 10 μM forward and reverse primer, 15 μl of 2 × Taq PCR Master Mix and 12 μl of deionised water. The ITS region (approximately 900 bp) was amplified using the universal primers ITS5 (GGAAGTAAAAGTCGTAACAAGG) and ITS4 (TCCTCCGCTTATTGATATGC) (White et al. 1990). The Cox1 gene ((approximately 700 bp) was amplified using the universal primers OomCoxI-Levlo (CYTCHGGRTGWCCRAAAAACCAAA) and OomCoxI-Levup (TCAWCWMGATGGCTTTTTTCAAC) (Robideau et al. 2011) . PCR conditions for ITS were as follows: initial denaturation at 95°C for 3 min, followed by 35 cycles of 94°C for 40 s, 54°C for 45 s and 72°C for 1 min and a final extension of 72°C for 10 min. PCR conditions for Cox1 were as follows: initial denaturation at 94°C for 3 min, followed by 35 cycles of 94°C for 30 s, 52°C for 30 s and 72°C for 1 min and a final extension of 72°C for 10 min (Rahman et al. 2014). PCR products were purified and sequenced by Genscript (Nanjing, China) using the same primers.

Sequences, generated in this study, were aligned with additional sequences downloaded from GenBank (Table 1) using ClustalX (Thompson et al. 1997) and manually adjusted in BioEdit (Hall 1999). The sequence alignment has been deposited in TreeBase (http://purl.org/phylo/treebase; submission ID S24209).

**Phylogenetic analyses**

Phylogenetic analysis was conducted as descibed by Cui et al. (2019). Maximum Likelihood (ML) and Bayesian Inference (BI) methods were also used to generate phylogenetic trees from the combined ITS and Cox1 dataset. Two isolates of *Saprolegnia parasitica* Coker were used as outgroups (Villa et al. 2006). Substitution models suitable for ITS partition and Cox1 partition of the dataset were determined using the Akaike Information Criterion implemented in MrMODELTEST2.3 (Nylander 2004). The General Time Reversible + proportion Invariant + Gamma (GTR+I+G) substitution model was selected for each partition. RAxML v.7.2.6 (Stamatakis 2006) was used for ML analysis. All parameters in the ML analysis used the default setting and statistical support values were obtained using non-parametric bootstrapping with 1000 replicates. A Bayesian tree was inferred using MrBayes3.1.2 (Ronquist and Huelsenbeck 2003), with a general time reversible model of DNA substitution and an invgamma distribution rate variation across sites. Eight Markov chains were run from the random starting tree for 2 million generations of the combined ITS and Cox1 dataset and sampled every 100 generations. Chain convergence was determined using Tracer v.1.5 (http://tree.bio.ed.ac.uk/software/tracer/) to confirm sufficiently large ESS values (> 200). The burn-in parameter was set to discard the first 25% of trees. A majority rule consensus tree of all remaining trees was generated for each analysis. Branches receiving bootstrap values for ML and Bayesian posterior probabilities (BPP) greater than or equal to 75% (ML) and 0.95 (BPP) were considered significantly supported. Phylogenetic trees were visualised using FigTree v.1.4.2 (http://tree.bio.ed.ac.uk/software/figtree/).

### Pathogenicity

Pathogenicity was confirmed using the hypocotyl slit inoculation method (Dorrance et al. 2008). Three-day-old V8A plugs (1.5 cm diam.) of isolate Chen 94 were used to infect the soybean cultivar 'Hefeng 47'. Five soybean seedings inoculated with uncolonised agar plugs served as controls. The inoculated soybean seedings (five plants for the isolate) were incubated at 25°C with a 12-h photoperiod in a greenhouse for 4–5 days. Experiments were conducted in triplicate.

## Taxon treatments

### Pythium huanghuaiense

Jia J. Chen & X.B. Zheng 2021 sp. n.

3F822E05-AFE4-554F-BCFD-394C117AF0E7

822954

#### Materials

- **Type status:** Holotype. **Occurrence:** recordedBy: Jiajia Chen; **Taxon:** scientificName: Pythium huanghuaiense; class: Oomycetes; order: Pythiales; family: Pythiaceae; genus: Pythium; **Location:** country: China; stateProvince: Jiangsu; locality: Nanjing, Jiangning District, Pengfu Village; **Identification:** identifiedBy: Jiajia Chen; **Event:** year: 2016; month: 4; day: 29; habitat: on seedlings of *Glycine max*; **Record Level:** type: chen 94 (BJFC-C 1993, metabolically inactive culture); language: en
- **Type status:** Paratype. **Occurrence:** recordedBy: Jiajia Chen; **Taxon:** scientificName: Pythium huanghuaiense; class: Oomycetes; order: Pythiales; family: Pythiaceae; genus: Pythium; **Location:** country: China; stateProvince: Jiangsu; locality: Nanjing, Jiangning District, Pengfu Village; **Identification:** identifiedBy: Jiajia Chen & Xiaobo Zheng; **Event:** year: 2016; month: 4; day: 1; habitat: on seedlings of *Glycine max*; **Record Level:** type: Chen 95 (NJAU-JN18, JAFLA 95; metabolically inactive culture) & Chen 96 (NJAU-JN19, JAFLA 96; metabolically inactive culture); language: en
- **Type status:** Paratype. **Occurrence:** recordedBy: Jiajia Chen; **Taxon:** scientificName: Pythium huanghuaiense; class: Oomycetes; order: Pythiales; family: Pythiaceae; genus: Pythium; **Location:** country: China; stateProvince: Anhui; locality: Suzhou; **Identification:** identifiedBy: Jiajia Chen & Xiaobo Zheng; **Event:** year: 2016; month: 7; habitat: on seedlings of *Glycine max*; **Record Level:** type: Chen 99 (NJAU-JN30, JAFLA 99; metabolically inactive culture); language: en
- **Type status:** Paratype. **Occurrence:** recordedBy: Jiajia Chen; **Taxon:** scientificName: Pythium huanghuaiense; class: Oomycetes; order: Pythiales; family: Pythiaceae; genus: Pythium; **Location:** country: China; stateProvince: Shandong; locality: Jining; **Identification:** identifiedBy: Jiajia Chen & Xiaobo Zheng; **Event:** year: 2016; month: 8; habitat: on seedlings of *Glycine max*; **Record Level:** type: Chen 100 (NJAU-JN65, JAFLA 100; metabolically inactive culture); language: en
- **Type status:** Other material. **Occurrence:** recordedBy: Jiajia Chen; **Taxon:** scientificName: Pythium huanghuaiense; class: Oomycetes; order: Pythiales; family: Pythiaceae; genus: Pythium; **Location:** country: China; stateProvince: Jiangsu; locality: Nanjing, Jiangning District, Pengfu Village; **Identification:** identifiedBy: Jiajia Chen & Xiaobo Zheng; **Event:** year: 2016; month: 4; day: 29; habitat: on seedlings of *Glycine max*; **Record Level:** type: JAFLA 94, NJAU-JN11 (isotypes, metabolically inactive culture); language: en

#### Description

**Figure 1.**
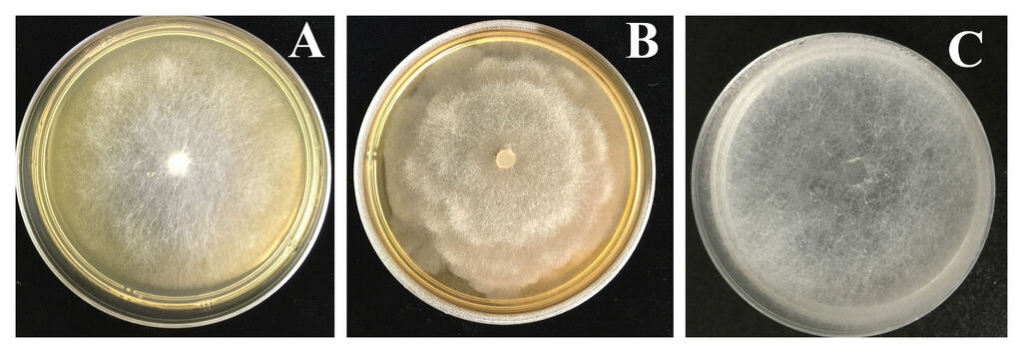
Colony patterns of *Pythium huanghuaiense* (Chen 94) on **A.** CMA; **B.** PCA; and **C.** V8A.

**Figure 2.**
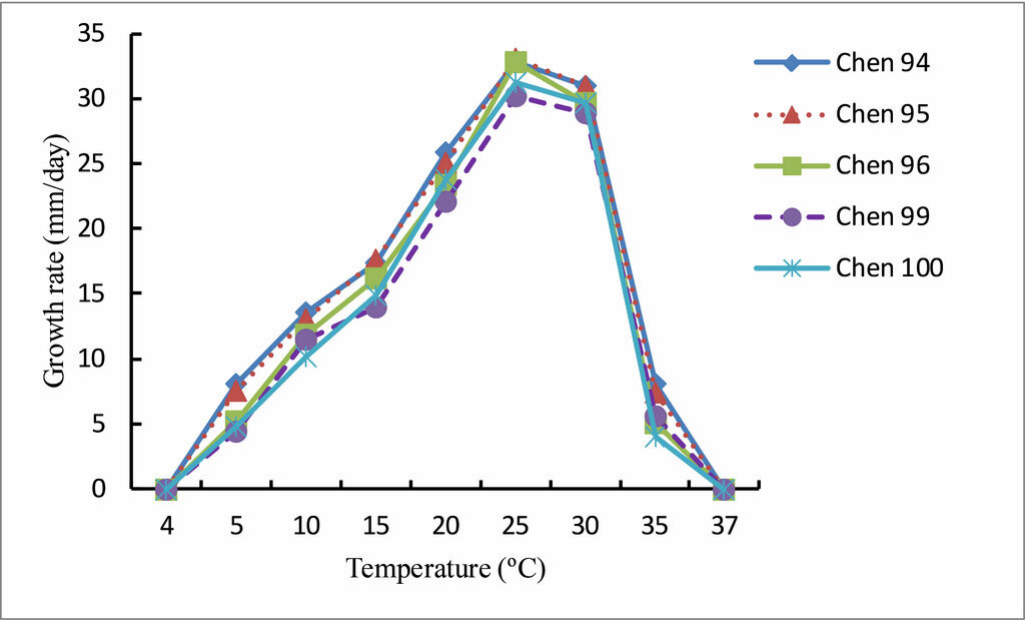
Mycelial growth rate of isolates of *Pythium huanghuaiense* Chen 94, 95, 96, 99 and 100 on PCA at different temperatures.

**Figure 3.**
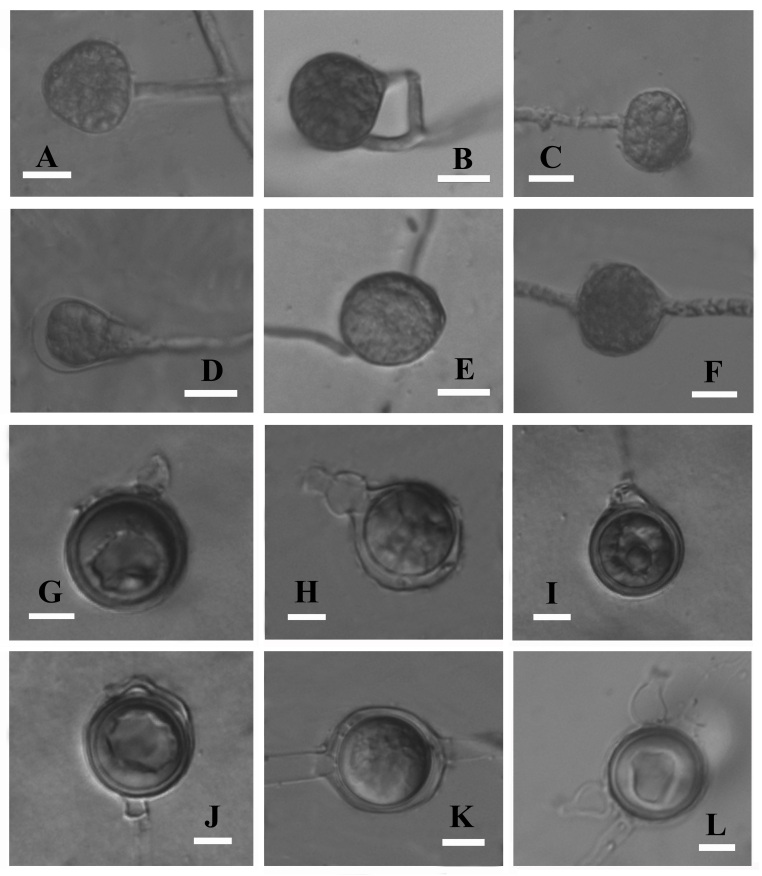
Asexual and sexual reproductive bodies of *Pythium huanghuaiense* (Chen 94). **A.** Obturbinate hyphal swelling; **B.** globose hyphal swelling; **C.** sub-globose hyphal swelling; **D.** pyriform hyphal swelling; **E, F.** intercalary hyphal swellings; **G, H.** oogonia with a projections; **I.** nearly plerotic oospore; **J.** elongated antheridial cell wavy in contour; **K.** intercalary oogonium; **L.** Nearly plerotic oospore and two antheridia. Bars: A–E 10 μm; G–J 5 μm.

Pathogenic on soybean. Colonies submerged, with a cottony pattern on CMA, a rosette pattern on PCA and a cottony pattern on 10% V8A (Fig. 1). Average growth rates of 32.8 mm/day at 25°C on PCA (Fig. 2). Cardinal temperatures: minimum 4°C, optimum 25°C, maximum 37°C. Main hyphae hyaline, aseptate, up to 5.0 µm wide. Hyphal swellings globose, sub-globose, obturbinate to pyriform, mostly terminal or sometimes intercalary, 15–22.5 × 13.5–20 (mean 19 × 17.5) μm. Sporangia and zoospores not observed. Homothallic; oogonia globose, smooth or with a projection, terminal or intercalary, 12.5–18 μm (mean 15.5 µm) in diameter. Antheridia mostly monoclinous, sometimes hypogynous, one to two per oogonium; antheridial stalks unbranched, arising at various distances from oogonia; antheridial cells sub-globose, club-shaped or fist-shaped, making broad or narrow apical contact with oogonia. Oospores plerotic or nearly plerotic, globose, 11.5–17 μm (mean 14.5 µm) in diameter, hyaline. Oospore wall 0.5–1.5 µm (mean 1.1 µm) thick (Fig. 3).

#### Etymology

With reference to the distribution of the species in the Huang-Huai area of China.

#### Notes

*Pythium huanghuaiense* can be distinguished morphologically from its closest relatives, including *P. mamillatum* Meurs, *P. paroecandrum* Drechsler, *P. spiculum* B. Paul and *P. wuhanense* Y.Y. Long, J.G. Wei & L.D. Guo, by its narrower hyphae and relatively higher maximum growth rate. Additional differences between the novel species and other related species are listed in Table 2.

## Analysis

### Isolates

Five cultures of *Pythium* (Chen 94–96, Chen 99 and Chen100), representing an unknown species of *Pythium*, were obtained from soybean plant samples collected from three fields in three cities during April and August 2016.

### Molecular phylogeny

Five ITS and Cox1 sequences were newly generated for this study and their accession numbers are available in GenBank (Table 1). BLAST analyses of the ITS and Cox1 sequences of the five isolates, described here as *Pythium huanghuaiense*, showed the best phylogenetic matches with species of clade F in *Pythium* (Lévesque and Cock 2004).

**Figure 4.**
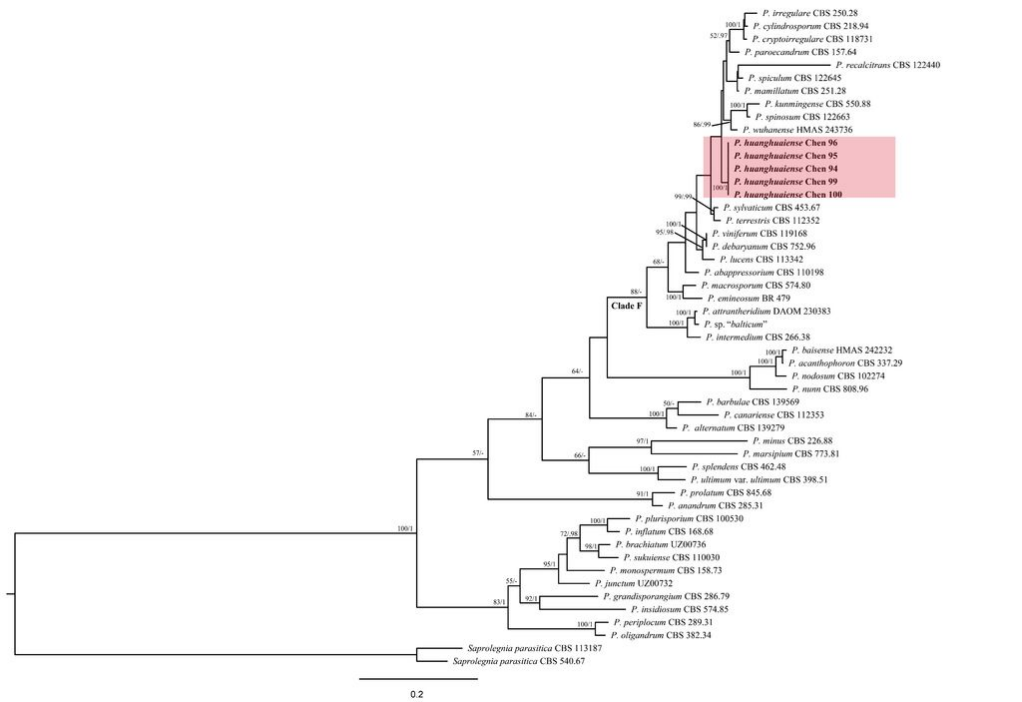
Phylogeny of *Pythium huanghuaiense* and related species generated by Maximum Likelihood, based on ITS+ Cox1 sequences. Branches are labelled with parsimony bootstrap proportions (before slanting line) higher than 50% and Bayesian posterior probabilities (after slanting line) more than 0.95. The branch of the new species is highlighted in pink.

ML and BI analyses yielded similar tree topologies and only the ML tree is shown (Fig. 4). The five isolates of the novel species, *P. huanghuaiense*, formed a well-supported lineage (100% ML and 1 BPP), indicating that they are phylogenetically distinct from other species of clade F in *Pythium* (Fig. 4).

### Pathogenicity

**Figure 5.**
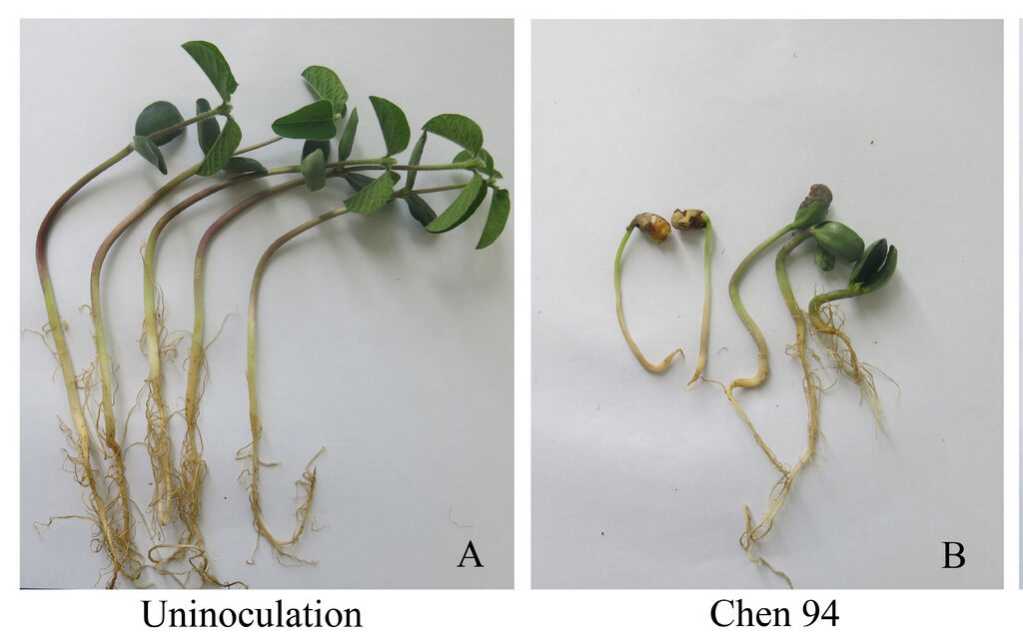
Pathogenicity of *Pythium huanghuaiense* (Chen 94) on the soybean cultivar Hefeng 47. **A.** Control; **B.** disease symptoms caused by *P. huanghuaiense*.

*Pythium huanghuaiense* (Chen 94) significantly stunted and reduced the growth of soybean seedlings compared with uninoculated controls (Fig. 5). To fulfil Koch’s postulates, pieces of diseased tissues obtained from inoculated plants were placed on V8A to re-isolate the causal agent. *Pythium huanghuaiense* could be recovered from the diseased soybean seedlings and was identified, based on morphological characteristics and comparisons of ITS and Cox1 sequences. According to Feng et al. (2020), pathogenicity tests, using dish cultures of *P. huanghuaiense* isolates and pots containing *P. huanghuaiense* cultures on soybean cultivar 'Zhonghuang 13', respectively, showed that *P. huanghuaiense* significantly reduced the germination rates of soybean and was highly pathogenic on this plant. These results confirm that *P. huanghuaiense* is a soybean pathogen with a high degree of pathogenicity.

## Discussion

*Pythium huanghuaiense* is characterised by globose, sub-globose, ellipsoid, obturbinate to pyriform hyphal swellings; smooth and relatively small oogonia (12.5–18 μm); mostly monoclinous, sometimes hypogynous antheridia; sub-globose, club-shaped or fist-shaped antheridial cells; and plerotic or nearly plerotic and thin-walled oospores (0.5–1.5 µm).

According to Lévesque and Cock (2004), *Pythium* can be split into 11 clades (A-K), of which clade F is composed of species with either globose, non-proliferating sporangia or globose hyphal swellings (only *P. irregulare* Buisman develops both) and a fast growth rate (often more than 25 mm/day; Lévesque and Cock 2004). Phylogenetic analysis, based on ITS and Cox1 sequences, indicated that *P. huanghuaiense* belongs to clade F of *Pythium* with full statistical support. *Pythium huanghuaiense* shares several morphological characteristics with other species of clade F, such as smooth oogonia and a fast growth rate. However, *P. huanghuaiense* can be readily distinguished from other species by having narrower hyphae and a relatively higher maximum growth rate.

*Pythium huanghuaiense* is similar to *P. wuhanense* in its quick growth. The two species are phylogenetically closely related, belonging to clade F of *Pythium* (Fig. 4), but the former has narrower hyphae and plerotic or nearly plerotic oospores (Long et al. 2014; Table 2). Both *P. mamillatum* and *P. spiculum* have similar sized oogonia and they share some similarity with *P. huanghuaiense*; however, these two species can be readily distinguished from *P. huanghuaiense* by the ornamentation on their oogonia (van der Plaäts-Niterink 1981, Paul et al. 2006;Table 2). In addition, these three species clustered in different lineages in the phylogenetic analysis. *P. huanghuaiense* differs from *P. paroecandrum* by its quicker growth rate, narrower hyphae and plerotic or nearly plerotic oospores (van der Plaäts-Niterink 1981).

Soybean is a major source of edible oil and protein and plays an important role in the human diet. Many species of *Pythium* are reported to be pathogens of soybean and some studies have documented the diversity of members of this genus, as well as their pathogenicity on soybean (such as Zhang and Yang 2000, Zitnick-Anderson and Nelson 2015, Coffua et al. 2016, Radmer et al. 2017). However, the diversity and importance of *Pythium* spp. as pathogens in China, particularly in soybean, are largely unknown. In a recent study on *Pythium* and *Phytopythium* spp. in a soybean–wheat rotation system in the Huang-Huai region, *P. huanghuaiense* (as an undescribed candidatus species) was highly pathogenic on soybean and wheat (Feng et al. 2020). As part of an ongoing study on the diversity of *Pythium* spp. associated with soybean in China, the novel species, *P. huanghuaiense*, was identified and described in this study on the basis of morphological characteristics and ITS and Cox1 sequence data. Additional pathogenicity tests and studies on the economic impact of *P. huanghuaiense* on soybean and other crop plants will be conducted in the future.

## Supplementary Material

XML Treatment for Pythium huanghuaiense

## Figures and Tables

**Table 1.** A list of species, cultures and GenBank accession numbers of sequences used in this study.

Species name	Sample no.	Locality	GenBank accession no.	
			ITS	*Cox1*
*Pythium abappressorium*	CBS 110198	USA	HQ643408	HQ708455
*P. acanthophoron*	CBS 337.29	USA	HQ643413	HQ708460
*P. alternatum*	CBS 139279	Japan	AB998876	AB998877
*P. anandrum*	CBS 285.31	–	HQ643435	HQ708482
*P. attrantheridium*	DAOM 230383	Canada	HQ643477	HQ708524
*P. baisense*	HMAS 242232	China	FR775440	FR774198
*P. barbulae*	CBS 139569	Japan	LC028389	LC028392
*P. brachiatum*	UZ00736	Japan	KJ995581	KJ995593
*P. canariense*	CBS 112353	Spain	HQ643482	HQ708528
*P. cryptoirregulare*	CBS 118731	USA	HQ643515	HQ708561
*P. cylindrosporum*	CBS 218.94	Germany	HQ643516	HQ708562
*P. debaryanum*	CBS 752.96	UK	HQ643519	HQ708565
*P. emineosum*	BR 479	UK	GQ244427	GQ244423
*P. grandisporangium*	CBS 286.79	USA	HQ643546	HQ708590
***P. huanghuaiense***	**Chen 94**	**China**	**MF984118**	**MF984155**
***P. huanghuaiense***	**Chen 95**	**China**	**MF984119**	**MF984156**
***P. huanghuaiense***	**Chen 96**	**China**	**MF984120**	**MF984157**
***P. huanghuaiense***	**Chen 99**	**China**	**MF984121**	**MF984158**
***P. huanghuaiense***	**Chen 100**	**China**	**MF984122**	**MF984159**
*P. inflatum*	CBS 168.68	USA	HQ643566	HQ708610
*P. insidiosum*	CBS 574.85	Costa Rica	HQ643570	HQ708614
*P. intermedium*	CBS 266.38	Netherlands	HQ643572	HQ708616
*P. irregulare*	CBS 250.28	Netherlands	HQ643596	HQ708640
*P. junctum*	UZ00732	Japan	KJ995576	KJ995595
*P. kunmingense*	CBS 550.88	China	HQ643672	HQ708716
*P. lucens*	CBS 113342	UK	HQ643681	HQ708725
*P. macrosporum*	CBS 574.80	Netherlands	HQ643684	HQ708728
*P. mamillatum*	CBS 251.28	Netherlands	HQ643687	HQ708731
*P. marsipium*	CBS 773.81	Netherlands	HQ643690	HQ708734
*P. minus*	CBS 226.88	United Kingdom	HQ643696	HQ708740
*P. monospermum*	CBS 158.73	United Kingdom	HQ643697	HQ708741
*P. nodosum*	CBS 102274	France	HQ643709	HQ708753
*P. nunn*	CBS 808.96	USA	HQ643711	HQ708755
*P. oligandrum*	CBS 382.34	United Kingdom	HQ643715	HQ708759
*P. paroecandrum*	CBS 157.64	Australia	HQ643731	HQ708772
*P. periplocum*	CBS 289.31	USA	HQ643743	HQ708784
*P. plurisporium*	CBS 100530	USA	HQ643749	HQ708790
*P. prolatum*	CBS 845.68	USA	HQ643754	HQ708795
*P. recalcitrans*	CBS 122440	Spain	DQ357833	EF426549
*P.* sp. "*balticum*"	CBS 122649	Sweden	HQ643478	HQ708525
*P. spiculum*	CBS 122645	France	HQ643790	HQ708831
*P. spinosum*	CBS 122663	India	HQ643791	HQ708832
*P. splendens*	CBS 462.48	USA	HQ643795	HQ708836
*P. sukuiense*	CBS 110030	Taiwan	HQ643836	HQ708877
*P. sylvaticum*	CBS 453.67	USA	HQ643845	HQ708886
*P. terrestris*	CBS 112352	France	HQ643857	HQ708898
*P. ultimum var. ultimum*	CBS 398.51	Netherlands	HQ643865	HQ708906
*P. viniferum*	CBS 119168	France	HQ643956	HQ708997
*P. wuhanense*	HMAS 243736	China	HE862398	HE862402
*Saprolegnia parasitica*	CBS 113187	Russia	HQ644005	HQ709046
*S. parasitica*	CBS 540.67	United Kingdom	HQ644000	HQ709041

New sequences are shown in bold.

**Table 2.** Morphological description of *Pythium huanghuaiense* and the most closely related species.

	*Pythium huanghuaiense* (Chen 94)	*P. mamillatum*	*P. paroecandrum*	*P. spiculum*	*P. wuhanense*
Width of hyphae (μm)	Up to 5	Up to 6.5	Up to 9	Up to 6	Up to 7.5
Sporangia/hyphal swellings	Globose, sub-globose, obturbinate to pyriform, mostly terminal or sometimes intercalary	Globose, broadly ovoid or ellipsoidal, intercalary or lateral	Globose or ellipsoidal, intercalary or terminal	Globose, ovoid, cylindrical and at times peanut-shaped, mostly intercalary to catenulate, rarely terminal	Globose, sometimes cylindrical to elongated, mainly intercalary, often catenulate with oogonia, occasionally terminal or lateral
Oogonia (μm)	12.5–18 (av. 15.5), terminal or intercalary	15–18 (av. 16), intercalary or terminal	17–24 (av. 19), intercalary, often in chains and rarely terminal	13–22 (av. 15.6), mostly intercalary or in chains	10–20 (av. 17.7), mostly intercalary, often catenulate with sporangia and antheridia, sometimes terminal or lateral
Oogonium ornamentation	Absent	Present	Absent	Present	Absent
Antheridia	Mostly monoclinous, sometimes hypogynous	Mostly monoclinous, infrequently diclinous	Mostly monoclinous, sometimes diclinous	Monoclinous	Monoclinous, hypogynous or diclinous
Oospores (μm)	Plerotic or nearly plerotic, 11.5–17 (av. 14.5)	Plerotic， 12–15 (av. 14)	Aplerotic， 15–21 (av. 17)	Plerotic or aplerotic， 8–18	Aplerotic， 7.5–17.5 (av. 14.5)
Oospore wall thickness (μm)	0.5–1.5	0.8–1.4	1–1.5	0.5–1	0.5–1
Double oospores	Absent	Absent	Absent	Present	Present
Cardinal temperature	Min 4°C, optimum 25°C and max 37°C	Min 5°C, optimum 25°C and max 30–35°C	Min 5°C, optimum 25°C and max 35°C	Unknown	Min 4°C, optimum 28–30°C and max 35°C
Daily growth rates on PCA at 25°C (mm)	32	25	20–25	25	50
Reference	This study	van der Plaäts-Niterink (1981)	van der Plaäts-Niterink (1981)	Paul et al. (2006)	Long et al. (2014)

## References

[B6757402] Ali-Shtayeh M. S., Saleh A. S.F. (1999). Isolation of *Pythium
acanthicum*, *P.
oligandrum*, and *P.
periplocum* from soil and evaluation of their mycoparasitic activity and biocontrol efficacy against selected phytopathogenic *Pythium* species. Mycopathologia.

[B6757434] Chen J. J., Feng H., Song W., Zheng X. B. (2020). Two new *Pythium* species from southern China based on morphological and molecular characters. Phytotaxa.

[B6757443] Coffua L. S., Veteran S. T., Clipman S. J., Mena-Ali J. I., Blair J. E. (2016). Characterization of *Pythium* spp. associated with asymptomatic soybeans in southeastern Pennsylvania. Plant Disease.

[B6757453] Cui B. K., Li H. J., Ji X., Zhou J. L., Song J., Si J., Yang Z. L., Dai Y. C. (2019). Species diversity, taxonomy and phylogeny of Polyporaceae (Basidiomycota) in China. Fungal Diversity.

[B6757466] Dorrance A. E., Berry S. A., Anderson T. R., Meharg C. (2008). Isolation, storage, pathotype characterization, and evaluation of resistance for *Phytophthora
sojae* in soybean. Plant Health Progress.

[B6757485] Feng H., Chen J. J., Yu Z., Li K., Li Z., Li Y. X., Sun Z., Wang Y. C., Ye W. W., Zheng X. B. (2020). Pathogenicity and fungicide sensitivity of *Pythium* and *Phytopythium* spp. associated with soybean in the Huang-Huai region of China. Plant Pathology.

[B6757510] Hall T. A. (1999). Bioedit: a user-friendly biological sequence alignment editor and analysis program for windows 95/98/NT. Nucleic Acids Symposium Series.

[B6757529] Ho H. H. (2013). The genus *Pythium* in mainland China. Mycosystema.

[B6757538] Lévesque C. A., Cock A. W.A.M. (2004). Molecular phylogeny and taxonomy of the genus *Pythium*. Mycological Research.

[B6757547] Long Y. Y., Wei J. G., Sun X., He Y. Q., Luo J. T., Guo L. D. (2012). Two new *Pythium* species from China based on the morphology and DNA sequence data. Mycological Progress.

[B6757558] Long Y. Y., Sun X., Wei J. G., Sun X., Wei J. J., Deng H., Guo L. D. (2014). Two new species, *Pythium
agreste* and *P.
wuhanense*, based on morphological characteristics and DNA sequence data. Mycological Progress.

[B6757570] Miller P. M. (1955). V-8 juice agar as a general purpose medium for fungi and bacteria. Phytopathology.

[B6757579] Nylander J. A.A. (2004). MrModeltest v2. Program distributed by the author.

[B6757616] Paul B., Bala K., Lassaad B., Calmin G., Sanchez-Hernandez E., Lefort F. (2006). A new species of *Pythium* with ornamented oogonia: morphology, taxonomy, internal transcribed spacer region of its ribosomal RNA, and its comparison with related species. FEMS Microbiology Letters.

[B6757627] Pringsheim N. (1858). Beiträge zur morphology and systematik der algen. 2. Die Saprolegníeen [Contributions to the morphology and systematics of algae]. Jahrbücher für Wissenschaftliche Botanik.

[B6757647] Radmer L., Anderson G., Malvick D. M., Kurle J. E. (2017). *Pythium*, *Phytophthora*, and *Phytopythium* spp. associated with soybean in Minnesota, their relative aggressiveness on soybean and corn, and their sensitivity to seed treatment fungicides. Plant Disease.

[B6757636] Rahman M. Z., Uematsu S., Coffey M. D., Uzuhashi S., Suga H., Kageyama K. (2014). Re-evaluation of Japanese *Phytophthora* isolates based on molecular phylogenetic analyses. Mycoscience.

[B6757680] Robideau G. P., Cock A. W., Coffey M. D., Voglmayr H., Brouwer H., Bala K., Désaulniers N., Chitty D. W., Eggertson Q. A., Gachon C. M., Hu C. H., Küpper F. C., Rintoul T. L., Sarhan E., Verstappen E. C., Zhang Y., Bonants P. J., Ristaino J. B., Lévesque C. A. (2011). DNA barcoding of oomycetes with cytochrome c oxidase subunit I and internal transcribed spacer. Molecular Ecology Resources.

[B6757704] Ronquist F., Huelsenbeck J. P. (2003). MRBAYES 3: Bayesian phylogenetic inference under mixed models. Bioinformatics.

[B6849604] Stamatakis A. (2006). RAxML-VI-HPC: maximum likelihood-based phylogenetic analyses with thousands of taxa and mixed models. Bioinformatics.

[B6757721] Thompson J. D., Gibson T. J., Plewniak F., Jeanmougin F., Higgins D. G. (1997). The Clustal_X windows interface: flexible strategies for multiple sequence alignment aided by quality analysis tools. Nucleic Acids Research.

[B6757731] Ueta S., Tojo M. (2016). *Pythium
barbulae* sp. nov. isolated from the moss, *Barbula
unguiculata*; morphology, molecular phylogeny and pathogenicity. Mycoscience.

[B6757740] Uzuhashi S., Okada G., Ohkuma M. (2015). Four new *Pythium* species from aquatic environments in Japan. Antonie van Leeuwenhoek.

[B6757758] van der Plaäts-Niterink AJ (1981). Monograph of the genus *Pythium*. Studies in Mycology.

[B6850129] Villa N. O., Kageyama K., Asano T., Suga H. (2006). Phylogenetic relationships of *Pythium* and *Phytophthora* species based on ITS rDNA, cytochrome oxidase II and tubulin gene sequences. Mycologia.

[B6757767] Wang P. H., Chung C. Y., Lin Y. S., Yeh Y. (2003). Use of polymerase chain reaction to detect the soft rot pathogen, *Pythium
myriotylum*, in infected ginger rhizomes. Letters in Applied Microbiology.

[B6757789] White T. J., Bruns T., Lee S., Taylor J. (1990). Amplification and direct sequencing of fungal ribosomal RNA genes for phylogenetics. PCR Protocols.

[B6757798] Wrather J. A., Koenning (2006). Estimates of disease effects on soybean yields in the United States 2003 to 2005. Journal of Nematology.

[B6757807] Zhang B. Q., Yang X. B. (2000). Pathogenicity of *Pythium* Populations from corn–soybean rotation fields. Plant Disease.

[B6757816] Zitnick-Anderson K. K., Nelson B. D. (2015). Identification and pathogenicity of *Pythium* on soybean in North Dakota. Plant Disease.

